# Ultrasensitive Protein Aggregate Quantification Assays for Neurodegenerative Diseases on the Simoa Platform

**DOI:** 10.1021/acs.analchem.4c04188

**Published:** 2024-12-24

**Authors:** Dorothea Böken, Zengjie Xia, Jeff Y. L. Lam, Emre Fertan, Yunzhao Wu, Elizabeth A. English, Juraj Konc, Florence Layburn, Gonçalo J. L. Bernardes, Henrik Zetterberg, Matthew R. Cheetham, David Klenerman

**Affiliations:** † Yusuf Hamied Department of Chemistry, University of Cambridge, Cambridge CB2 1EW, U.K.; ‡ UK Dementia Research Institute at University of Cambridge, Cambridge CB2 0XY, U.K.; § Department of Psychiatry and Neurochemistry, Institute of Neuroscience and Physiology, The Sahlgrenska Academy at the University of Gothenburg, Mölndal 43139, Sweden; ∥ Clinical Neurochemistry Laboratory, Sahlgrenska University Hospital, Mölndal 43180, Sweden; ⊥ Department of Neurodegenerative Disease, UCL Institute of Neurology, Queen Square, London WC1N 3BG, U.K.; # UK Dementia Research Institute at UCL, London W1T 7NF, U.K.; ¶ Hong Kong Center for Neurodegenerative Diseases, Hong Kong 999077, China; ∇ Wisconsin Alzheimer’s Disease Research Center, University of Wisconsin School of Medicine and Public Health, University of Wisconsin–Madison, Madison, Wisconsin 53792, United States

## Abstract

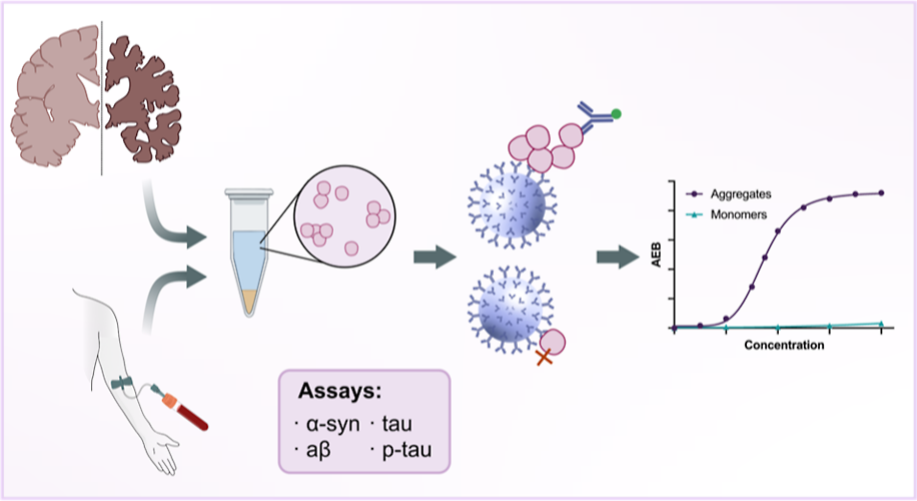

Nanoscale aggregates play a key role in the pathogenesis of neurodegenerative disorders such as Alzheimer’s and Parkinson’s disease. However, quantifying these aggregates in complex biological samples, such as biofluids and postmortem brain tissue, has been challenging due to their low concentration and small size, necessitating the development of methods with high sensitivity and specificity. Here, we have developed ultrasensitive assays utilizing the Quanterix Simoa platform to detect α-synuclein, β-amyloid and tau aggregates, including those with common posttranslational modifications such as truncation of α-synuclein and AT8 phosphorylation of tau aggregates. All assays had a detection limit in the low pM range. As a part of this work, we developed silica-nanoparticle calibrators, allowing for the quantification of all aggregates. These assays were validated for aggregate and target specificity through denaturation and cross-reactivity experiments. We then applied these assays to brain homogenate samples from Alzheimer’s disease and control samples, demonstrating their applicability to postmortem tissue. Lastly, we explored the potential of these assays for blood-based diagnostics by detecting aggregates in serum samples from early Alzheimer’s disease patients.

The formation and aggregation of amyloids play an important role in the pathogenesis of neurodegenerative diseases.^1,2^ In Alzheimer’s disease (AD), β-amyloid (Aβ) aggregates form extra-cellular plaques and hyperphosphorylated tau aggregates form intracellular tangles.^3^ In Parkinson’s disease (PD), α-synuclein (α-syn) aggregates form intracellular inclusions called Lewy bodies.^4^ While these larger, insoluble aggregates are commonly studied in AD and PD, a body of work suggests that the smaller (submicron), soluble aggregates formed during the aggregation process are the primary cytotoxic species, initiating and promoting the pathology through a variety of mechanisms.^5−8^ It is therefore important to develop methods to detect small aggregates in accessible biofluids, for diagnosis of disease, and also in tissue samples to study disease mechanisms. However, this is challenging due to the low concentration of these aggregates in biofluids and the occurrence of significant posttranslational modifications^9,10^ during the aggregation process such as truncation and phosphorylation, which may hinder immunolabeling.^11−13^

It is possible to detect the presence of fibrillar, seeding-competent aggregates by protein-seed amplification-based methods (RT-QuIC).^14^ To more generally detect aggregates present in a sample, the majority of methods are based on antibody capture of the aggregate and then sandwiching it with a second antibody, which could be either directly labeled with a fluorophore (immunofluorescence) or coupled to an enzyme (ELISA). In the case of immunofluorescence, aggregates are then detected by counting the number of fluorescent spots,^15^ therefore the accuracy of these methods depends on the image resolution (restricted by the diffraction limit) and the algorithm used to identify the particles. In the case of ELISA, total enzyme activity is detected,^16^ reflecting the number of antibodies bound to the captured aggregates. Other methods used for detection have included the use of impedance to measure the total amount of monomeric protein before and after denaturation of the aggregates.^17^ These techniques highlight the challenges and considerations involved in accurately detecting and quantifying protein aggregates.

There are two main challenges associated with these types of assays to detect protein aggregates. First, ensuring that the assays specifically detect aggregates and not monomers, since the monomer concentration is often much higher than the aggregate concentration and monomers are not toxic;^18^ and second, reducing nonspecific binding of the imaging antibody to enable sensitive detection of the low concentrations of aggregates. The first issue is generally addressed by using the same monoclonal antibody with a single epitope, for capture and detection, which means that captured monomer will not give rise to a signal. Detection antibodies labeled with two different fluorophores have also been used to selectively identify aggregates by detection of coincident signals.^19^ The second issue of nonspecific binding is commonly addressed by using a passivated surface, blocking steps, and multiple washing steps.

To date, a number of ELISA-based methods have been developed to detect aggregates. Two α-syn oligomer-specific kits are commercially available with sensitivities above 50 pg/mL. Similarly, two Aβ aggregate specific ELISAs have been developed,^20,21^ one of which has sufficient sensitivity to detect the low level of aggregates in cerebrospinal fluid (CSF).^21^ There is also one total tau aggregate specific kit available, but none for phosphorylated tau aggregates. However, ELISA-based methods for aggregate quantification measure total aggregate mass, not concentration. Measurements of both aggregate size and number in brain samples, CSF and serum, using superresolution imaging^7,8,22^ show that the aggregates are present in a range of sizes with a large number of small aggregates, which are the main contributor to the aggregate concentration and a small number of large aggregates which are the main contributor to the aggregate mass. Therefore, while ideally one would want to measure both the aggregate number and size distribution to characterize the aggregates present, both aggregate mass and concentration are important and more tractable aggregate properties to measure. Furthermore, since smaller aggregates are the toxic species in many neurodegenerative diseases it is important to be able to measure aggregate concentration.^5−8^

To address this problem, several single-molecule immunoassays have been developed in recent years, including surface-based fluorescence intensity distribution analysis (sFIDA) and single molecule array (Simoa). sFIDA is a fluorescence-microscopy based technique, developed to detect Aβ, α-syn, and tau aggregates.^23,24^ Similar to ELISA, sFIDA captures analytes to form immunocomplexes on a surface but then reports the number of pixels showing fluorescence intensity above a set threshold. As part of this work, calibration samples based on monomer coupled to silica beads were developed, allowing the signal to be converted to an approximate concentration.^25,26^ These silica beads can be bound by multiple antibodies and hence serve as model aggregates of similar size to the aggregates that we previously detected in serum.^27^ However, sFIDA has only been optimized for Aβ aggregates in complex biological matrices, such as serum and plasma,^28,29^ meanwhile tau and α-syn aggregates have only been detected in CSF.

The Simoa assay, developed and commercialized by Quanterix Inc. (Figure 1A),^30^ uses antibody-coated beads for capture of the target of interest in a given sample which are then incubated with biotinylated detector antibody. An immunocomplex is formed with streptavidin β-galactosidase (SBG), which can generate a fluorescent readout through resorufin β-d-galactopyranoside (RGP). The Simoa assay is highly sensitive for low concentrations, as the beads will only have one or zero immunocomplexes on it, enabling a digital readout.

**Figure 1:**
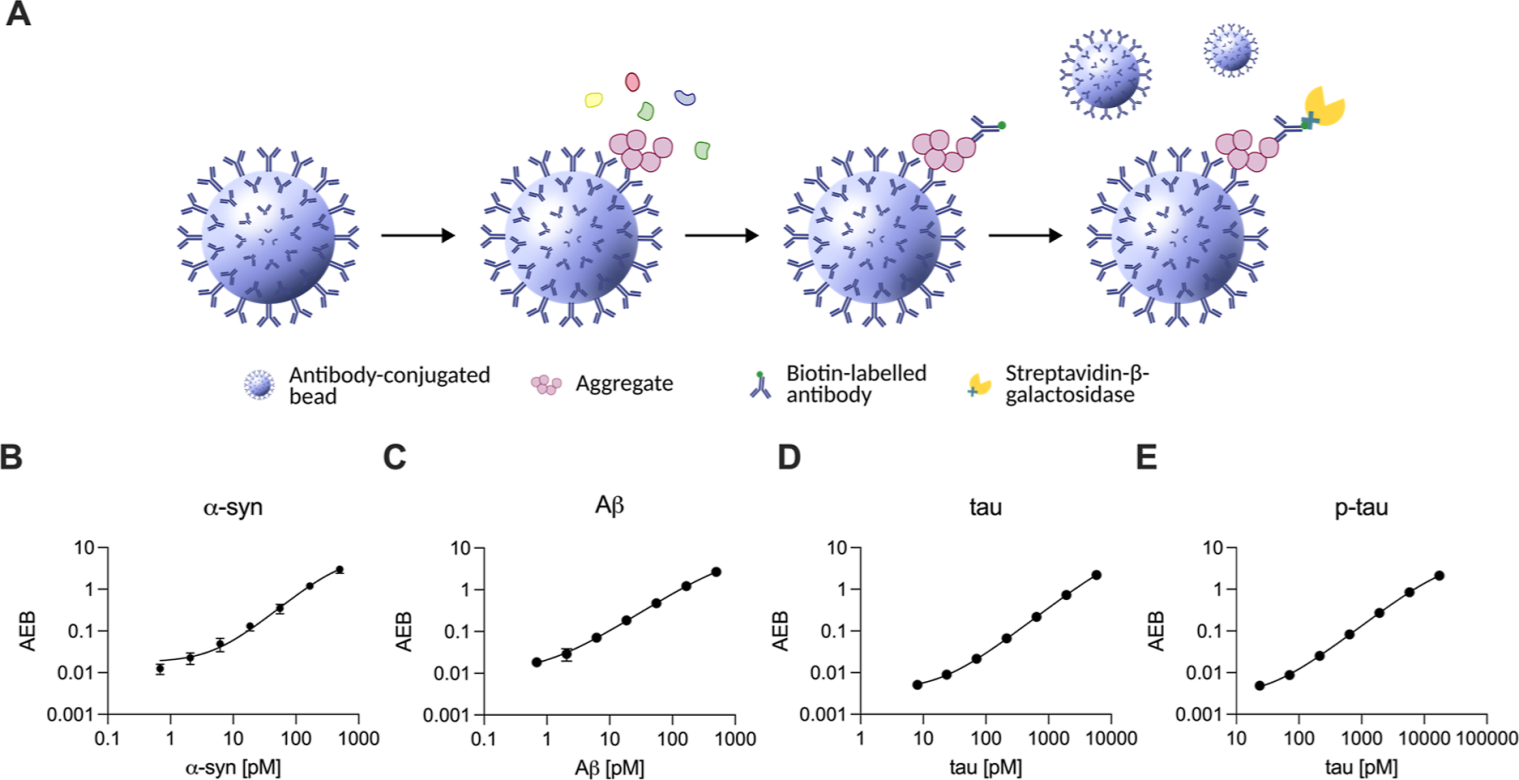
Simoa for the detection of protein aggregates. (A) Schematic representation of the Simoa assay for aggregate detection using the same monoclonal antibody for capture and detection to ensure aggregate detection. Antibody-conjugated paramagnetic beads are used to capture protein aggregates present in a sample. Upon the addition of biotin-labeled antibody an immunocomplex is formed, which can be bound by streptavidin-β-galactosidase, capable of generating fluorescent product. At low concentrations, each bead captures a maximum of a single aggregate. (B) Standard curve of α-syn calibrator using the 4B12–4B12 antibody pair. (C) Standard curve of Aβcalibrator using the 6E10–6E10 antibody pair. (D) Standard curve of tau lysate calibrator using the HT7–HT7 antibody pair. (E) Standard curve of tau lysate calibrator using the AT8–AT8 antibody pair. Panel B–E show the mean of *n* = 4 technical replicates.

While protein aggregates play a critical role in disease pathology, they are typically present at very low concentrations, posing a challenge for detection using conventional methods.^7,8,22^ Simoa presents a promising platform for ultrasensitive aggregate-specific assays, surpassing the detection limits of conventional immunoassays. So far, Simoa has predominantly been utilized for detecting total protein (monomeric + aggregated) but presents a promising platform for ultrasensitive aggregate-specific assays. Currently existing Simoa-based aggregate assays lack the required sensitivity to detect aggregates in clinical samples, such as the tau assay which only recognizes epitopes for the microtubule binding region and not those covering the N- or C-terminal region, nor relevant posttranslational modifications.^31^ The aim of this work was to develop a Simoa-based assay with high enough sensitivity and specificity allowing the detection of α-syn, Aβ, and tau aggregates as well as aggregates containing disease-relevant posttranslational modifications in biological samples including tissue and biofluids.

## Results

### Establishing Simoa Assays for the Selective Detection of Protein Aggregates

We set out to develop Simoa aggregate assays to detect protein aggregates relevant to neurodegenerative diseases, including native and C-terminal truncated α-synuclein (α-syn), β-amyloid (Aβ), tau, and phosphorylated tau (p-tau). For the specific detection of these aggregates—and not monomers—we used the same monoclonal antibody for both capture and detection (Figure 1A). This configuration required the presence of two or more identical epitopes within a single aggregate; capturing the aggregate would occupy one binding site and require a second site for binding of the detection antibody, ensuring that the detected species are, at a minimum, dimeric. The assay development process consisted of antibody pair selection, assay condition optimization (detector concentration and SBG concentration), optimization to achieve lower coefficient of variation at all calibration levels including adjusting the dynamic range, assay validation, and diluent optimization for specific sample matrices.

To achieve accurate quantification and ensure normalization between runs, we first developed suitable calibrators for these assays. This is a challenging task since the calibrator needs to have a known concentration and be reproducible. Since the aggregation process of monomers is stochastic and the concentration of the aggregates produced is unknown, in vitro aggregates are not suitable calibrators. To have a reliable aggregate mimic we coupled α-syn, Aβ42, or tau monomers to silica nanoparticles. We used single-molecule and superresolution microscopy to check if the calibrators aggregate (Figure S1A–F). We did not observe any subpopulation of larger calibrator aggregates using diffraction-limited nor superresolution microscopy, making the coated silica nanoparticles suitable calibrators for our purposes. Since it is not possible to get a sample of tau in which every single monomer is phosphorylated at S202 and T205 (AT8), the silica nanoparticle-based calibrators could not be used for p-tau. To solve this issue, we used cell lysate from HEK cells which stably propagate tau aggregates that are highly phosphorylated.^22^ This allowed us to use the same sample for measurement of total tau aggregates and tau aggregates phosphorylated at S202 and T205 (AT8-positive).

It should be noted the obtained concentrations are an approximation, as the exact number of silica nanoparticles in the calibrator samples is not known due to potential small losses in their synthesis.

We then proceeded to select the antibody pairs for each assay. For the development of the α-syn assays we selected the 4B12–4B12 and sc12767–sc12767 (SC211) antibody pairs based on their target specificity (truncated and total, respectively) and limits of detection. It is known that significant truncation of α-syn occurs during the pathogenesis of PD, increasing aggregation.^32^ Importantly, this truncation can remove the epitope detected by the SC211 antibody, which is closer to the C-terminus.^33^ Use of the 4B12 antibody, which has an epitope closer to the NAC region of α-syn, allows us to detect truncated α-syn along with full-length α-syn. By detecting both we can estimate the concentration of truncated α-syn aggregates. For the tau assay, we selected two antibodies that are commonly used in the field: the Ser202 and Thr205 phosphorylated tau-specific antibody AT8^34^ and the total tau antibody HT7.^35^ For the Aβ assay, we used the 6E10 antibody, which has a binding region within residues 3–8 of Aβ.^36^ All five antibodies selected (4B12, SC211, 6E10, HT7, AT8) are monoclonal and have a single epitope, and as such only bind to a single epitope on each monomer. We ensured that using these antibody pairs we were able to detect the respective silica-nanoparticle and lysate calibrators explained above, generating a concentration-dependent signal which can be used as calibration curves for each assay (Figure 1B–E).

**Figure 2:**
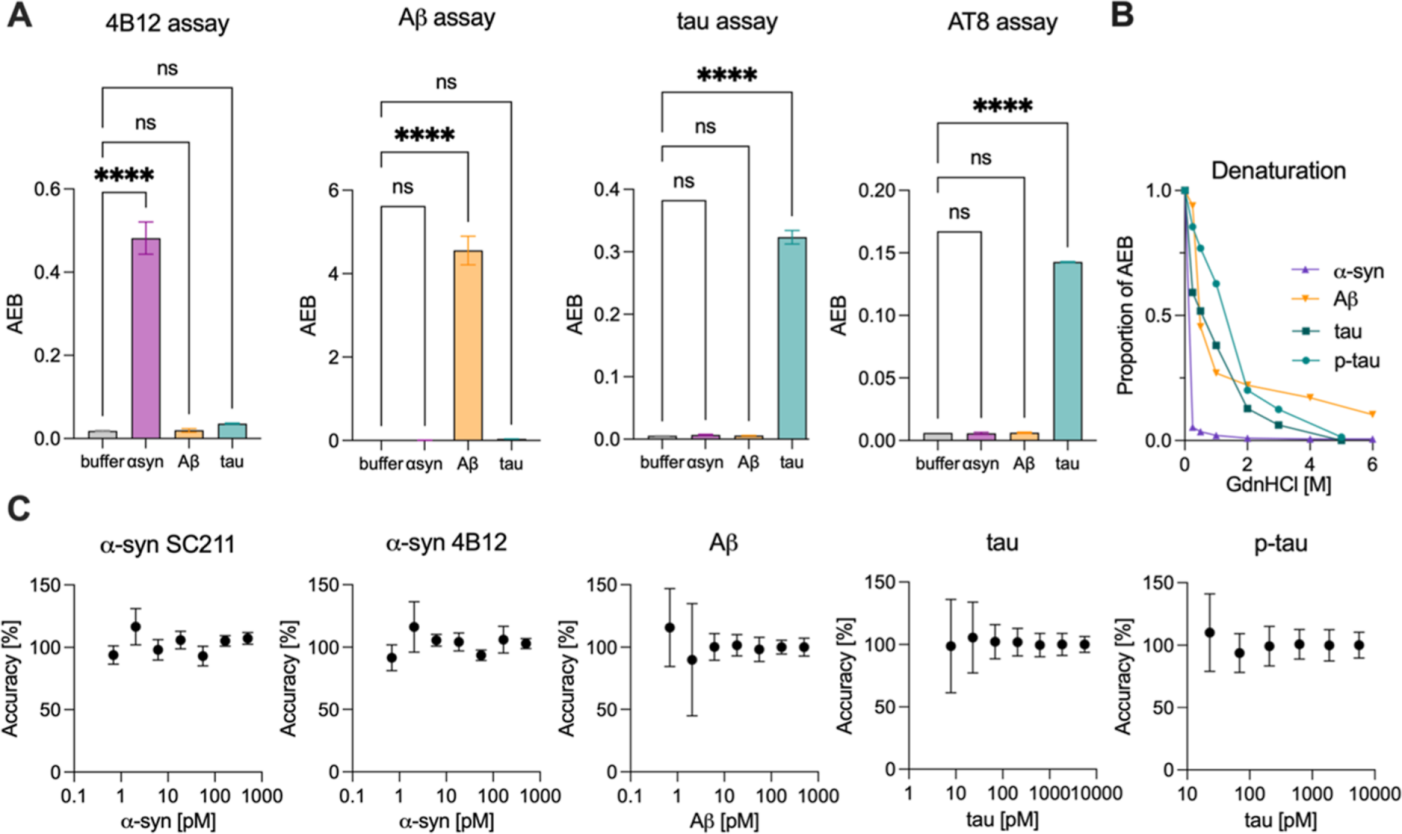
Validation of Simoa assays for the detection of protein aggregates. (A) Simoa assays were tested for cross-reactivity against other protein aggregates to ensure specificity for the respective aggregate. (B) Denaturation of the aggregates with increasing concentrations of guanidinium chloride (0–6 M) to test the specificity of the Simoa assays for protein aggregates as opposed to monomers. (C) Accuracy of the aggregate assays across the working range for α-syn aggregates (4B12 antibody pair), Aβ aggregates (6E10 antibody pair), tau aggregates (HT7 antibody pair), p-tau aggregates (AT8 antibody pair). Statistical analysis was conducted using one-way ANOVA and Tukey’s multiple comparisons test. Panel A shows the mean ± SD of *n* = 3, B of *n* = 2, and C of *n* = 4 technical replicates. ns: *p* > 0.05, ****: *p* < 0.0001.

To ensure the accuracy of our protein aggregate quantification assays, we evaluated specificity from two key perspectives. First, the assay must be specific to the analyte of interest. While the specificity largely depends on the antibodies used, it can also be influenced by other reagents involved in the assay. To test this, we assessed the cross-reactivity between Aβ, α-syn, and tau aggregates by measuring the recombinant aggregate samples using mismatched Simoa assays. The results showed negligible cross-reactivity, confirming high analyte-specificity (Figure 2A). Second, the assay must specifically detect aggregates rather than monomer. To evaluate this, we performed denaturation experiments of the aggregates with increasing concentrations of guanidinium chloride (GdnHCl), and a short heat treatment (85 °C for 10 min). Following denaturation, 99% of the signal disappeared showing that the assays are aggregate specific (Figure 2B).

To further enhance assay performance, we optimized the sample diluent, detector, and SBG concentration for each assay individually, evaluating performance based on the signal-to-background ratio at various calibration levels (Figure S2A). Initially, we identified the optimal sample diluent by testing a range of commercially available diluents (Quanterix Sample Diluents A–E), alongside the standard sample diluent and, specifically for the tau aggregate assays, the tau 2.0 sample diluent. The standard diluent is phosphate buffer with saline, surfactant and bovine serum components. The Quanterix sample diluents are A, phosphate buffer with bovine serum components, a heterophilic blocker, and a surfactant; B, phosphate buffer with protein stabilizers (bovine), a heterophilic blocker, and a high surfactant concentration; C, phosphate buffer with low concentration of protein stabilizers (bovine), a heterophilic blocker, and a surfactant; D, phosphate buffer with newborn calf serum, a heterophilic blocker, and a surfactant; E, Tris buffer with high pH, bovine serum components, a heterophilic blocker, and a surfactant. The tau 2.0 diluent contains BSA, calf serum and an antimicrobial.

For the detector and SBG enzyme concentrations, we explored various combinations of their concentrations together to account for potential combined effects (Figure S2B). Using the optimized assay conditions, we achieved the following limits of detection (LoDs): 4.2 pM for 4B12 assay, 0.63 pM for SC211, 0.92 pM for 6E10, 17 pM for HT7, 37 pM for AT8 (Table 1). The optimal assay conditions and respective LoDs are shown in Tables S1 and 1, the signal-to-noise ratio across the dynamic range in Figure S3A.

**Table 1:** Comparison of the Limit of Detection (LoD) of the Simoa Aggregate-Specific Assays

aggregate assay	antibody	LoD (pM)
α-syn truncated	4B12	4.2
α-syn total	SC211	0.625
Aβ	6E10	0.92
tau	HT7	17
p-tau	AT8	37

To confirm the reproducibility of the assays, we validated their accuracy and precision using four technical replicates across 2 days using calibrator samples as well as independently prepared quality control samples across the calibration range. All assays had a coefficient of variation (CV) below the accepted 20% threshold^37^ throughout the working range. The accuracy of all assays laid between 80% and 120% for all calibration samples (Figure 2C) as well as for independently prepared quality control samples (*n* = 5 technical replicates on each plate) at multiple concentrations selected in the dynamic range of the respective assay (Figure S3C,D).

### Detection of Soluble Aggregates in the Human Brain

Once the assays were optimized, we applied them to postmortem human brain homogenate samples to demonstrate their capabilities to detect soluble protein aggregates in relevant biologically complex samples. Given the critical role of these aggregates in AD,^38,39^ postmortem brain homogenate from AD and age-matched control samples was tested. We first ensured that these assays detect aggregates in a concentration-dependent manner in a linear range. For this purpose, brain homogenate from 5 AD and 5 control samples were combined and tested across a wide range of dilutions (α-syn 1:4 to 1:256, Aβ 1:6.25 to 1:400, tau: 1:2000 to 1:64,000, Figure S4A). We confirmed the linear working range of the assays for brain homogenate by interpolating the protein aggregate concentration from the standard curves and calculating the final concentration of the aggregates depending on the dilution factor, observing only little variation across the dilution range (Figure S4B). This positive correlation of the brain homogenate concentration and the readout confirms the detection of aggregates in this sample type.

**Figure 3:**
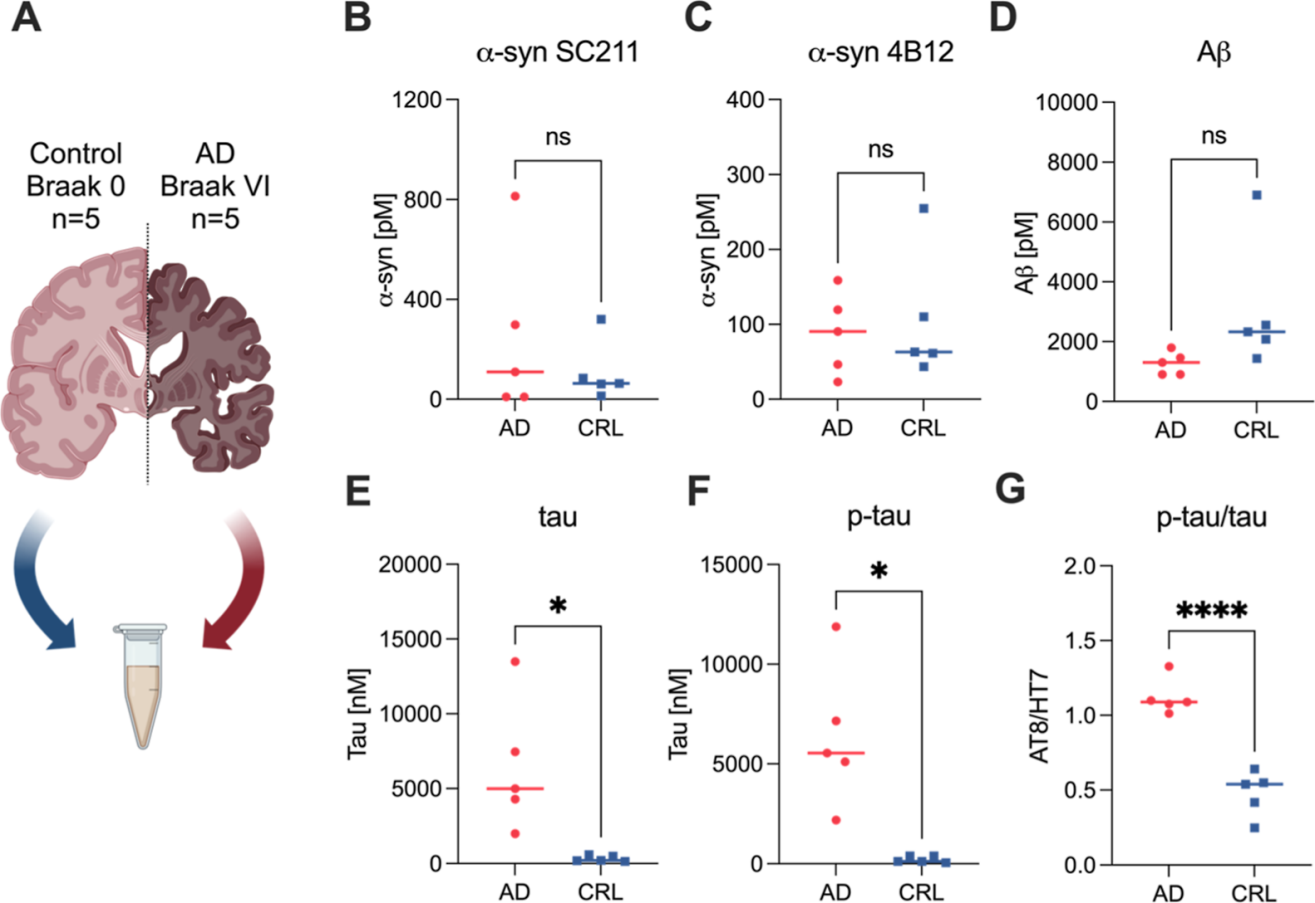
Aggregate levels in brain homogenate from AD and control patients. (A) Schematic of postmortem brain tissue samples used in the study. (B–G) Aggregate levels detected in brain homogenate from AD (frontal cortex, Braak Stage VI) and control patients (frontal cortex, Braak Stage 0) using aggregate Simoa assays for (B) α-synuclein aggregates (SC211 antibody pair), (C) α-synuclein aggregates (4B12 antibody pair), (D) Aβ aggregates (6E10 antibody pair), (E) tau aggregates (HT7 antibody pair), (F) p-tau aggregates (AT8 antibody pair). (G) The ratio of p-tau to total tau aggregates was determined showing improved separation between AD and control patients. Each data point in the plot represents the mean of 2 technical replicates. Panel A–G show the mean of *n* = 5 AD and *n* = 5 control patients from *n* = 2 technical replicates. Statistical analysis was conducted using Welch’s *t*-test. ns: *p* > 0.05, *: *p* < 0.05, ****: *p* < 0.0001. Figure A was created using BioRender.com.

We then proceeded to test brain homogenate from 5 AD (frontal cortex, BA6/8, Braak Stage VI) and 5 age-matched controls (frontal cortex, BA6/8, Braak Stage 0; Figure 3A, Table S2). Using the calibrator samples to calculate the aggregate concentration, all samples were above zero, showing the detection of aggregates in these samples. Notably, we saw a significant increase in tau and p-tau aggregates with AD (Figure 3B–G). The mean concentration of α-syn aggregates detected by SC211 was 109 and 248 pM (SC211 assay, Welch’s *t*-test, *p* = 0.42) and 107 and 88 pM for aggregates detected by 4B12 (Welch’s *t*-test, *p* = 0.69), respectively in control and AD brains (Figure 3B,C), with no significant differences between control and disease. The Aβ concentration was 3060 pM in control brain and 1272 pM in AD (Welch’s *t*-test, *p* = 0.14, Figure 3D). This difference may reflect that there is a higher number of small Aβ aggregates in control brain and there is a small number of large aggregates in AD brain. The total tau (HT7) and AT8-positive aggregate concentration were 6446 and 6372 pM respectively in AD brains, compared to 328 and 218 pM in control brain (Welch’s *t*-test, HT7: *p* = 0.023, AT8: *p* = 0.018, Figure 3E,F). Thus, there were significantly increased levels of aggregated tau in AD brain. Overall, these results show that the dominant protein aggregate present in AD brain is tau. The ratio of AT8/HT7 aggregates was ∼1 in AD and <0.5 in controls, showing that the majority of aggregates detected in AD were phosphorylated at S202 and T205 (Welch’s *t*-test, *p* < 0.0001, Figure 3G).

### Aggregate Detection in Human Serum

**Figure 4:**
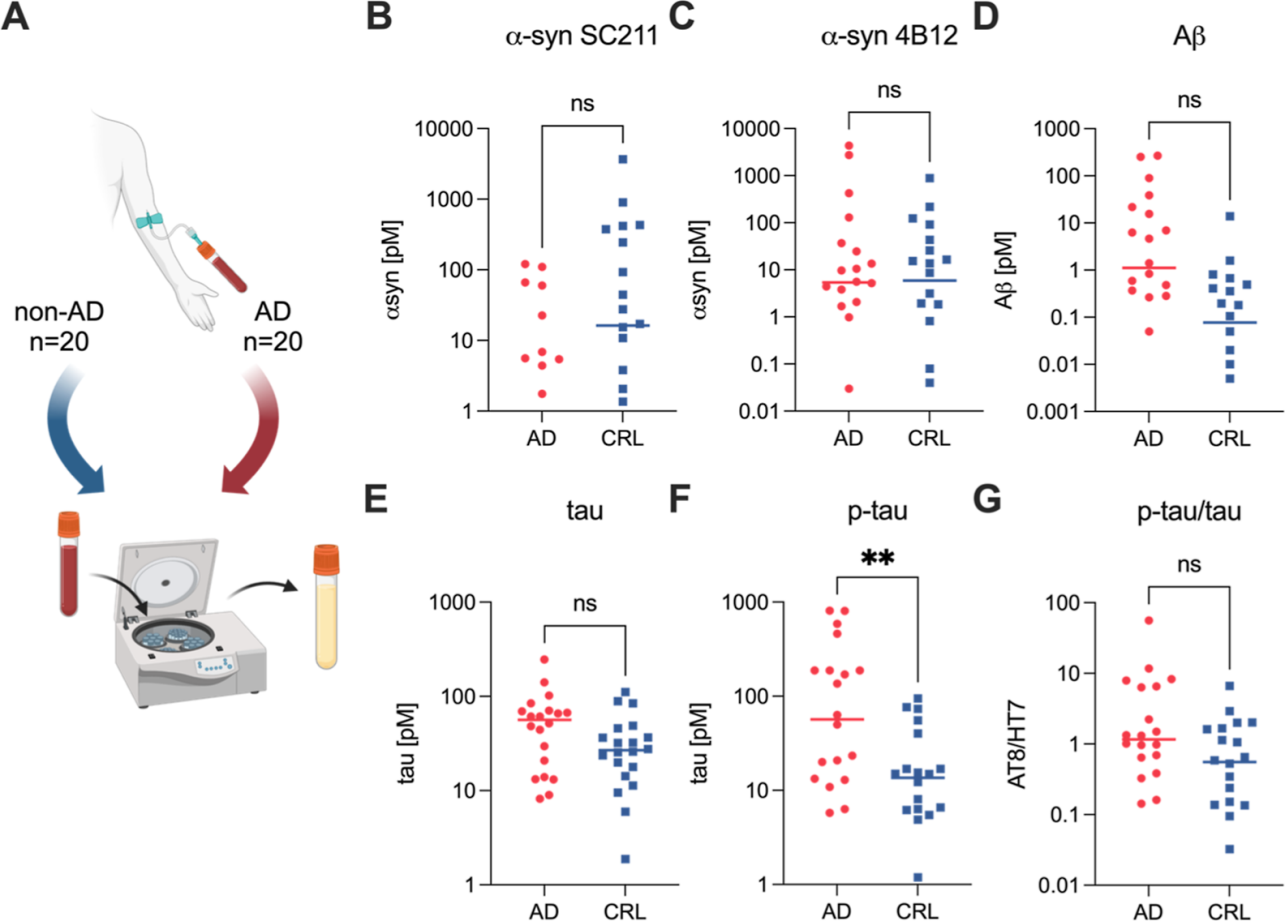
Quantification of protein aggregates in human serum. (A) Schematic of serum samples used in this study. Patients with early AD includes patients who sought medical advice at a memory clinic for the first time and were diagnosed for AD based on the positive AD CSF biomarker profile. (B–F) Aggregate levels detected in the serum of early AD and control patients using aggregate Simoa assays for (B) α-synuclein aggregates (SC211 antibody pair, 15 data points not shown on graph due to values being zero), (C) α-synuclein aggregates (4B12 antibody pair), (D) Aβ aggregates (6E10 antibody pair), (E) tau aggregates (HT7 antibody pair), (F) p-tau aggregates (AT8 antibody pair). (G) Ratio of p-tau to total tau aggregates. Each data point in the plot represents the mean of 2 technical replicates. Panel B–G show the mean ± SD of *n* = 20 AD and *n* = 20 control patients. Statistical analysis was conducted using a *t*-test. ns: *p* > 0.05, **: *p* < 0.01. Figure A was created using BioRender.com.

We last confirmed the assays are compatible with readily available and clinically relevant samples, such as human serum. The high concentration of albumin along with other proteins and lipids creates a complex matrix that may affect the diffusion and detection of the aggregates in serum. To address this, we optimized the detector and SBG concentrations specifically for serum to detect α-syn, Aβ, and tau aggregates (Figure S5A,B). Similar to the brain homogenate we verified the detection of aggregates in a linear range by testing a range of concentrations, observing a positive correlation between serum concentration and signal (Figure S5C). We then proceeded to analyze samples from patients from memory clinics in the Region of Västra Götaland, Sweden, diagnosed with early stage AD based on a positive CSF biomarker profile (*n* = 20, Figure 4A, Tables S3 and S4). Patients who were negative for this biomarker profile were used as the control group (*n* = 20).

While α-syn aggregate levels did not differ between AD and control (Figure 4B,C), there was a trend for higher Aβ (Figure 4D) and total tau (Figure 4E) in the CSF biomarker positive cases accompanied by significantly higher levels of AT8-positive tau aggregates (Figure 4D). Moreover, we observed 10-fold higher levels of total (C-terminally truncated + full length α-syn) aggregates than full length only aggregates, suggesting that most of the α-syn aggregates in these samples are C-terminally truncated. The ratio of AT8-positive tau aggregates to total tau aggregates was 3.1 and 0.67 for AD and control serum, respectively (Figure 4G), suggesting higher phosphorylation of tau in early AD.

While α-syn aggregate levels did not differ between AD and control (Figure 4B,C), there was a trend for higher Aβ (Figure 4D) and total tau (Figure 4E) in the CSF biomarker positive cases accompanied by significantly higher levels of AT8-positive tau aggregates (Figure 4D). Moreover, we observed 10-fold higher levels of total (C-terminally truncated + full length α-syn) aggregates than full length only aggregates, suggesting that most of the α-syn aggregates in these samples are C-terminally truncated. The ratio of AT8-positive tau aggregates to total tau aggregates was 3.1 and 0.67 for AD and control serum, respectively (Figure 4G), suggesting higher phosphorylation of tau in early AD.

## Discussion

We have developed quantitative, highly sensitive, and selective Simoa-based assays for detecting aggregates of α-synuclein (α-syn), β-amyloid (Aβ) and tau with detection limits in the low picomolar range. We confirmed the specificity for detecting aggregates only (and not monomers) and showed that the assays are not cross-reacting with other protein aggregates. Additionally, our assays can detect key posttranslational modifications, including C-terminally truncated α-syn and AT8-phosphorylated tau aggregates. We performed extensive bioanalytical validation to show the reproducibility and stability of all our assays.

These assays have been optimized for use in a wide range of complex biological samples, including human serum, postmortem brain tissue, as well as model systems such as cerebral organoid conditioned media^40^ and mouse models. The high sensitivity of our assays is crucial, as small, soluble aggregate levels in these samples are typically very low and often fall below the detection limits of other techniques. This sensitivity allows for the reliable detection of aggregates even in challenging biological matrices. This versatility makes our assays a valuable tool for studying disease mechanisms across various biological contexts. By applying these assays to different sample types, the assays can be used to gain insights into the aggregation processes and posttranslational modifications occurring in vivo and in vitro, enhancing our understanding of neurodegenerative disease progression.

A key feature of our method is the development of robust calibrators. These calibrators enable normalization between assay runs to mitigate effects of assay variability and enable accurate quantification of the aggregates. This is crucial for ensuring consistency and reliability in longitudinal studies and comparisons across different experimental setups. It is important to note that recombinant protein aggregates formed in vitro comprise a dynamic, heterogeneous group of aggregate species with vast size distributions, which may be sensitive to subtle changes in incubation or storage conditions. Meanwhile, silica bead-based calibrators are more homogeneous in size and hence are more suitable for calibration purposes. These assays achieved an LoD in the low pM range which is a necessity for testing biological samples since the concentration of pathological aggregates is low in these samples.

We demonstrated the capabilities of our assays to detect soluble aggregates in biological samples by applying them to human brain tissue. Postmortem brain homogenate from AD samples was first tested at different dilutions providing concentration-dependent readouts. This was a proof of principle for detection of all the aggregates in this sample type and helped determining the linear range. Then we compared the AD brain homogenates with age- and sex-matched control samples. The decision of measuring all the aggregates in the same sample was made to allow their direct comparison. As expected, the concentration of α-syn aggregates did not differ between AD and control brains even though in both cases above background levels were detected. This is in line with previous findings that soluble α-syn aggregates are present in brains without PD pathology.^7,41^ Agreeing with our earlier findings using different methods, total and AT8-positive soluble tau aggregate levels were significantly higher in AD brains.^22^ Interestingly, soluble Aβ aggregate concentrations did not differ between AD and control samples, which may be due to existing aggregates growing into larger soluble aggregates in later disease stages, as we previously observed in CSF.^6^ As such, these assays can detect α-syn, Aβ and tau aggregates in human postmortem brain samples which will enable them to be used in future studies providing novel insights into the molecular mechanisms underlying neurodegenerative diseases. Indeed, these assays can be used to study in frontotemporal dementia and amyotrophic lateral sclerosis, and even nonneurodegenerative diseases such as cancer (Wu et al.).^47^ This approach of detecting small soluble aggregates with high sensitivity and specificity also holds potential for investigating the efficacy of therapeutic interventions aimed at reducing or modifying protein aggregates in various neurodegenerative conditions.

We then explored the application of our assays to human serum which is an accessible (noninvasive) biofluid that can be used for the diagnosis of dementia and to track its progression. Serum samples from individuals who visited a memory clinic for the first time and showed a positive CSF biomarker profile were compared to samples from patients who also visited the memory clinic but showed a negative CSF biomarker profile. The high analytical sensitivity of the assays allowed us to use less than 150 μL of sample in total to test for all the aggregates, making them suitable for samples with limited availability and enabling the application of multiple assays to each patient. Similar to human brain homogenate, we showed a concentration-dependent readout for all aggregates showing the capability of the assays to detect α-syn, Aβ and tau aggregates in human serum samples. Overall, cases classified as AD by the CSF biomarker profile, had significantly higher levels of AT8-positive tau aggregates and a strong trend for higher total tau (CI_95_ = −2.62, 54.65) and Aβ (CI_95_ = −2.83, 71.54) aggregate levels. Once we summed the concentrations of Aβ, HT7 and AT8 aggregates for each patient the difference was significant (CI95 = 70.61, 378.97). It is needed to test the validity of these results with a larger cohort. Nevertheless, these results show the potential for the diagnostic value of our assays.

In conclusion, we have developed a robust and sensitive method for detecting α-syn, Aβ and tau aggregates, including posttranslationally modified forms, in human brain homogenate and serum. It should be noted that the techniques and calibrators described here are not limited to the antibodies we used in this study. We selected these antibodies for their common usage in the field of dementia research, but any monoclonal antibody can be applied. Even though our assays achieved low LoDs, these can be further improved if the primary goal is sensitivity. The LoD depends on the off-rate of the antibody^42^ as a slow off-rate means that more captured target remains on the bead and can therefore be detected. Furthermore, our assays are capable of detecting aggregates with posttranslational modifications in biological samples. Our results demonstrate the potential of this method for understanding how aggregates change with AD progression and for early disease diagnosis. This approach is also broadly applicable to other protein aggregates and other diseases.

## Materials and Methods

### Aggregate Preparation

Wild type α-syn samples were expressed, purified in *Escherichia coli* and stored at −80 °C as previously described^43^ and kindly provided by the Centre for Misfolding Diseases (CMD) at the University of Cambridge. In vitro Aβ42 one-week sonicated aggregates^5^ and cell-derived tau aggregate samples^22^ were prepared as previously described. The total tau concentration was determined through ELISA, which is an upper limit for the amount of tau aggregates and used this sample as calibration standard. By measuring the HEK cell lysate using the tau silica bead calibrant using the HT7 aggregate, we found that 0.1 ng/mL total tau monomer corresponds to 5.8 nM tau aggregates. We ensured that the Venus tag does not interfere with the 488 nm dye-labeled beads by testing 750 nm dye-labeled beads (Figure S6A) and determined the total tau aggregate concentration in the cell lysate using tau silica bead calibrant (Figure S6B,C).

### Homogenization of Postmortem Brain Tissue

The postmortem brain tissue samples were obtained from the Edinburgh Brain Bank, where they were flash-frozen and stored at −80 °C. The brain tissue samples were homogenized as previously described.^22^

### α-Synuclein and Aβ42 SiNaPs

α-Synuclein and Aβ42 SiNaPs were prepared following the protocol described by Herrmann and colleagues.^25^

### Tau SiNaPs

30 nm triethoxylpropylaminosilane silica nanoparticles (Merck) were incubated with NHS-activated carbonylacrylic reagent^44^ and *N*,*N*-diisopropylethylamine (DIPEA) in DMF (overnight, 37 °C, 200 rpm, in dark). The beads were washed with DMF and water, and incubated with tris(2-carboxyethyl)phosphine hydrochloride (TCEP) and RP hTAU (InVivo BioTech Services Gmbh) in Tris buffer (overnight, 37 °C, 200 rpm). The beads were washed, sonicated for 10 min and resuspended in 1:1 H_2_O/DMSO solution to give 10 μM, and stored at −20 °C until use.

### Synthesis of NHS-Activated Carbonylacrylic Reagent

To 3-benzoylacrylic acid and *N*-hydroxysuccinimide in anhydrous THF (purified as reported by Pangborn et al.^45^) *N*,*N*′-dicyclohexylcarbodiimide was added, stirred at 0 °C for 1 h and kept in a freezer (−20 °C) overnight. *N*,*N*-Dicyclohexylurea was removed by filtration. The crude product (70% yield) was isolated as a yellow solid after recrystallization form isopropanol (40 mL). NMR spectra were recorded on Bruker 400-Avance III HD, Avance DPX-400, 400-QNP Cryoprobe (400.1 MHz for ^1^H) in DMSO-*d*_6_ (Figure S7). ^1^H NMR data are in accordance with previous reports.^46^

### Single-Molecule Pull-Down

Single-molecule Pull-down (SiMPull) experiments were performed as previously described.^30^ Briefly, functionalized coverslips were incubated with NeutrAvidin, washed, incubated with biotinylated capture antibody (α-syn: SC211, Aβ: 6E10, tau: HT7). After a further wash, the diluted silica-nanoparticle calibrators were incubated for 1 h at RT or overnight at 4 °C. Subsequently, the wells were washed, blocked with BSA and fluorescently labeled detection antibody (α-syn: SC211, Aβ: 6E10, tau: HT7) was added, followed by a further wash. Imaging was performed on a home-built total internal reflection fluorescence (TIRF) microscope. Superresolution imaging was performed using STORM.

### Antibodies

The following antibodies were used in this study: SC211 (Santa Cruz, Cat. no. SC767), 4B12 (BioLegend, Cat. no. 807-808), 6E10 (BioLegend, Cat. no. 803007), HT7 (Thermo Fisher Scientific, Cat. no. MN1000), AT8 (Thermo Fisher Scientific, Cat. no. MN1020).

### Simoa Plate Preparation

Antibody-bead conjugation was performed as described in the Quanterix Homebrew instruction manual (further explained in Supporting Information). Simoa plates were prepared in a “3-step-assay” following the Quanterix protocol, using the conditions listed in Table S1. The final plate was processed on the Quanterix SR-X Instrument. We avoid any sample or calibrant concentrations where *f*_ON_ > 0.7 in order to remain in a digital regime where the readout depends only on the number of fluorescent beads, rather than their brightness (analog mode).

### Simoa Data Analysis

The preliminary limit of detection (LoD) for each assay was determined by the parameter A with a set multiplier (normally 1.3) and fitted back to the curve to get the LoD in units of concentration. The lower limit of quantification (LLoQ) was the lowest valid calibration level with coefficient of variation (% CV) less than 25%. For the validation of each assay, four plates were run across 2 days using calibrator samples as well as independently prepared quality control samples across the calibration range. The quality control samples were analyzed against the calibration curve, and the obtained concentrations were compared with the nominal value to obtain the accuracy as a percentage of the nominal value. The accuracy was determined within a single plate (within-run accuracy) as well as across different plates (between-run accuracy).

### Denaturation

Aggregates were diluted in PBS to 500 nM of Aβ or tau, or 17.5 nM of α-syn, with different concentrations of guanidine hydrochloride (GdnHCl, 0 to 6 M). The mixture was heated to 80 °C for 10 min, and immediately diluted to at least 100-fold with appropriate sample diluent and kept on ice.

## Supporting Information Available

Supporting figures and tables. Detailed protocol of antibody-bead conjugation, plate preparation and Simoa processing by Quanterix. Detailed protocol of aggregate sample preparation (PDF)
