# Correction to “Ultrasensitive Protein Aggregate Quantification Assays for Neurodegenerative Diseases on the Simoa Platform”

**DOI:** 10.1021/acs.analchem.5c00703

**Published:** 2025-02-11

**Authors:** Dorothea Böken, Zengjie Xia, Jeff Y. L. Lam, Emre Fertan, Yunzhao Wu, Elizabeth A. English, Juraj Konc, Florence Layburn, Gonçalo J. L. Bernardes, Henrik Zetterberg, Matthew R. Cheetham, David Klenerman

In the original version of the paper the values of tau aggregate concentrations quoted in the text from Figure 3 had the wrong units. The units in the figure were correct. The text should read:

“The total tau (HT7) and AT8-positive aggregate concentrations were 6446 and 6372 nM, respectively, in AD brains, compared to 328 and 218 nM in control brain (Welch’s *t*-test, HT7: *p* = 0.023, AT8: *p* = 0.018, Figure 3E,F).”

