# Supplementary material for: Ultrasensitive Protein Aggregate Quantification Assays for Neurodegenerative Diseases on the Simoa Platform
Source: Anal Chem. 2024 Dec 24;97(1):290–9. doi: 10.1021/acs.analchem.4c04188 (PMC11740166; doi:10.1021/acs.analchem.4c04188)
Supplement: Supplementary file 1 — ac4c04188_si_001.pdf [file ac4c04188_si_001.pdf]

Supplementary Information

Ultra-sensitive protein aggregate quantification assays for neurodegenerative diseases on the Simoa platform

Dorothea Böken[1,2]‡, Zengjie Xia[1,2]‡, Jeff Y. L. Lam[1,2], Emre Fertan[1,2], Yunzhao Wu[1,2], Elizabeth A. English[1,2], Juraj Konc[1], Florence Layburn[1,2], Gonçalo J. L. Bernardes[1], Henrik Zetterberg[3,4,5,6,7,8], Matthew R. Cheetham[1,2,5], David Klenerman[1,2]*

1. Yusuf Hamied Department of Chemistry, University of Cambridge, Cambridge CB2 1EW, UK.

2. UK Dementia Research Institute at University of Cambridge, Cambridge CB2 0XY, UK.

3. Department of Psychiatry and Neurochemistry, Institute of Neuroscience and Physiology, the Sahlgrenska Academy at the University of Gothenburg, Mölndal 43139, Sweden.

4. Clinical Neurochemistry Laboratory, Sahlgrenska University Hospital, Mölndal 43180, Sweden.

5. Department of Neurodegenerative Disease, UCL Institute of Neurology, Queen Square, London WC1N 3BG, UK.

6. UK Dementia Research Institute at UCL, London W1T 7NF, UK.

7. Hong Kong Center for Neurodegenerative Diseases, Hong Kong, China.

8. Wisconsin Alzheimer's Disease Research Center, University of Wisconsin School of Medicine and Public Health, University of Wisconsin-Madison, Madison, WI 53792, USA.

*Corresponding authors Email:

dk10012@cam.ac.uk

Table of Contents

Detailed experimental protocol for aggregate and sample preparation	S3
Detailed experimental protocol for Simoa	S5
Supplementary Figure S1	S7
Supplementary Figure S2	S8
Supplementary Figure S3	S9
Supplementary Figure S4	S10
Supplementary Figure S5	S11
Supplementary Figure S6	S12
Supplementary Figure S7	S13
Supplementary Table S1	S14
Supplementary Table S2	S15
Supplementary Table S3	S16
Supplementary Table S4	S17

Detailed experimental protocol for aggregate and sample preparation

Recombinant α-syn aggregates. Wild type α-syn samples were expressed, purified in *E. coli* and stored at -80°C as previously described[43] and kindly provided by the Centre for Misfolding Diseases (CMD) at the University of Cambridge. To remove pre-aggregation seeds, the solution was centrifuged at 91000 g at 4°C for 1 h by an ultracentrifuge (Optima TLX Ultracentrifuge, Beckman). The concentration of the supernatant was then determined by A280 (ε 280 = 5960 M −1 cm −1). The supernatant was then diluted to 70 µM in 1x PBS supplemented with 0.01% NaN_3 and incubated at 37°C with shaking at 200 rpm for 12 h or 48 h. The aggregates were aliquoted (20 µL) to avoid multiple freeze-thaw cycles. The aliquots were then snap-frozen and stored at -80°C.

In vitro Aβ42 one-week sonicated aggregates. Lyophilised monomeric recombinant Aβ42 peptide (Stratech, Cat. No. A-1170-2-RPE-1.0mg) was dissolved in PBS (pH = 7.4) at 200 µM on ice. The solution was quickly aliquoted and snap-frozen. To prepare recombinant Aβ42 fibrils, an aliquot was thawed and diluted to 4 µM in 1xPBS supplemented with 0.01% NaN_3 and incubated at 37 °C under quiescent conditions for one week. The Aβ42 fibril was then sonicated as described previously[44] with modification. The one-week aggregated Aβ42 aliquot was immersion sonicated in an ice water bath with a 3-mm-titanium probe (Sonicator microprobe 4422, Qsonica) mounted on a tip sonicator (Ultrasonic processor Q125, QSonica) at 20 kHz with 40% of power for 24×5-s bursts with 15-s rests between bursts. Thereafter, the sonicated aggregate was centrifuged, aliquoted (50 µL) and snap-frozen. The aliquots were stored at -80 °C until use.

Preparation of cell-derived tau. HEK293 cells expressing tau P301S-Venus[45] under the CMV promoter (pcDNA3) were maintained in DMEM supplemented with 10% FCS, 100 U/mL penicillin, 100 µg/mL streptomycin and grown at 37 °C and 5% CO2. The cells were seeded with 50 nM heparin-assembled recombinant 6xHis-tau P301S assemblies in the presence of 1% Lipofectamine2000 and a single clone was isolated (R1E5) that stably propagates tau P301S-Venus aggregates. Cells were lysed in lysis buffer (1x PBS, 1% w/v Triton X-100, 1x cOmplete™, EDTA-free Protease Inhibitor Cocktail mix, 1x PhosSTOP™ phosphatase inhibitor mix) on ice for 30 min. The lysate was then centrifuged at 14,000 × g for 15 min at 4 °C and the clarified lysate was aliquoted and stored at -20 °C. The total tau concentration was determined through ELISA, which is an upper limit for the amount of tau aggregates and used this sample as calibration standard. By measuring the HEK cell lysate using the tau silica bead calibrant using HT7 antibodies, which bind pan-tau, for capture and detection, we found that 0.1 ng/mL total tau monomer corresponds to 5.8 nM tau aggregates so that the conversion factor from 1 ng/mL to nM is 58. We ensured that the Venus tag does not interfere with the 488nm dye-labelled beads by testing 750nm dye-labelled beads (**Supplementary Figure S6A**) and determined the total tau aggregate concentration in the cell lysate using tau silica bead calibrant (**Supplementary Figure S6B, C**).

α-synuclein and Aβ42 SiNaPs were prepared following the protocol described by Herrmann and colleagues[25]. In brief, 11.47 µM in dimethylformamide (DMF), Merck, Cat. No. 660450) and 500 µL of 18.2-MΩ cm water was added to 500 µL of carboxylated silica nanoparticles (cSiNaP) (particle size with 15-nm diameter. The mixture was then centrifuged (10,000 g, room temperature, 1 h) and the pellet was resuspended with 500 µL of MES buffer (2-(*N*-morpholino)ethanesulfonic acid) buffer, 10 mM, pH 5.7). Meanwhile, 1-ethyl-3-(3-dimethylaminopropyl) carbodiimide (EDC, ThermoFisher, Cat. No. A35391) and sulfo-*N*-hydroxysuccinimide (sulfo-NHS, ThermoFisher, Cat. No. A39269) were freshly dissolved in cold MES buffer (10 mM, pH 5.7) at 52.16 mM (10 mg/mL) and 92.11 mM (20 mg/mL), respectively. The cSiNaP was then diluted to 100 nM in MES buffer. EDC and sulfo-NHS were then introduced into the diluted cSiNaP suspension such that the mixture contained 400 µM EDC and 100 µM sulfo-NHS. The reaction mixture was sonicated for 30 min and then centrifuged (10,000 g, room temperature, 1 h). The pellet, i.e. activated SiNaP, was resuspended in fresh 10 mM MES buffer to give 200 nM suspension. Meanwhile, the monomeric α-synuclein or Aβ42 peptide was then diluted in 10 mM MES buffer to give 20 µM solution. To 1 mL of the activated SiNaP suspension, 1 mL of diluted α-synuclein or Aβ42 solution was introduced. The reaction mixture was placed on a revolver rotator for overnight incubation at room temperature. Finally, it was centrifuged (10,000 g, 4 °C, 1 h) and the pellet was redispersed in 1 mL of 1:1 H_2O:DMSO (v/v). The suspension was sonicated for 10 min and then centrifuged (5,000 g, 4 °C, 1 h). The α-synuclein-conjugated SiNaP was then redispersed in 400 µL of 1:1 H_2O:DMSO (v/v) to give a 500 nM (based on the SiNaP) suspension, and stored at -20 °C until use.

Tau SiNaPs. To the 30-nm triethoxylpropylaminosilane silica nanoparticles (nSiNaP, Merck, Cat. No. 791334, 16.59 µM in water, 500 µL), 500 µL of DMF was introduced. The mixture was firstly centrifuged (10,000 g, room temperature, 1 hour) and resuspended with 500 µL of DMF. Meanwhile, the NHS-activated carbonylacrylic reagent[46] was dissolved in DMF to give a 1 M stock solution. *N,N*-diisopropylethylamine (DIPEA, Merck, Cat. No. D125806) was diluted in DMF to give a 1 M stock solution. To 5.6 µL of 1 M DIPEA solution, 96.4 µL of the bead suspension was introduced, followed by the addition of 5.6 µL of 1 M carbonylacrylic linker solution. The reaction was incubated overnight at 37 °C with shaking at 200 rpm in dark. The reaction mixture was centrifuged (15,000 g, room temperature, 15 min) and the pellet was resuspended in 500 µL of DMF. The suspension was then centrifuged at (15,000 g, room temperature, 15 min) and the pellet was resuspended with 96.4 µL of water. Meanwhile, a 10x Tris buffer (500 mM Tris, pH 8.5) was

prepared. Tris(2-carboxyethyl)phosphine hydrochloride (TCEP, Merck, Cat. No. 75259) was dissolved in water to give a concentration of 100 mM and the pH was adjusted to 7-8. To the pellet suspension, 60 µL of 10x Tris buffer and 3.2 µL of TCEP solution were added, followed by the addition of 0.5 mL of RP hTAU solution (InVivo BioTech Services Gmbh, 32 µM in 100 mM Tris, 150 mM NaCl and 1 mM EGTA). The reaction was incubated overnight at 37 °C with shaking at 200 rpm. Next, the reaction mixture was centrifuged (15,000 g, room temperature, 15 min) and the pellet was resuspended in 500 µL of 1:1 H_2O:DMSO solution (v/v). The mixture was then sonicated for 10 min and centrifuged (15,000 g, room temperature, 15 min). The pellet was resuspended in 160 µL of 1:1 H_2O:DMSO solution (v/v) to give 10 µM, and stored at -20 °C until use.

Synthesis of NHS-activated carbonylacrylic reagent. All reagents were purchased from commercial suppliers and used as received. THF was purified as reported by Pangborn et al.[47], solvent was pre-dried over sodium wire and then distilled from calcium hydride and lithium aluminium hydride. Merck Silica gel 60 was used for the flash column chromatography. Monitoring of reactions was performed using TLC Silica gel 60 F254 plates. Compounds were detected using shortwave (254 nm) UV lamp or by staining with an indicated solution prepared by known procedures. NMR spectra were recorded on Bruker 400-Avance III HD, Avance DPX-400, 400-QNP Cryoprobe (400.1 MHz for ^{1}H) in DMSO-*d6* (referenced to the residual solvent signal). Chemical shifts are given in ppm (δ-scale), coupling constants (*J*) in Hz.

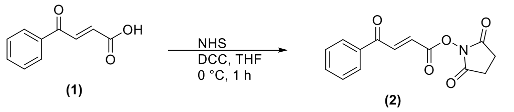

2,5-Dioxopyrrolidin-1-yl (*E*)-4-oxo-4-phenylbut-2-enoate (2)

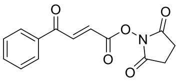

A solution of 3-benzoylacrylic acid (**1**) (1 g, 5.68 mmol) and *N*-hydroxysuccinimide (690 mg, 6.0 mmol) in anhydrous THF (25 mL) was cooled to 0°C and *N,N'*-dicyclohexylcarbodiimide (1.24 g, 6 mmol) was added with stirring. The reaction mixture was stirred at 0°C for 1 h and then it was kept in a freezer (-20°C) overnight. The formed *N,N*-dicyclohexylurea was removed by filtration, washed with ether and solvents were removed under reduced pressure. The product **2** (1.084 g, 4.0 mmol, 70% yield) was isolated as a yellow solid after recrystallization form isopropanol (40 mL). R_f 0.93 (10% MeOH/DCM, detection with UV light or $KMnO_4$ staining). ^{1}H NMR (400.1 MHz, DMSO-*d6*) δ 8.29 (d, *J* = 15.7 Hz, 1H, H3), 8.10–8.07 (m, 2H, H2''), 7.76–7.72 (m, 1H, H4''), 7.62–7.53 (m, 2H, H3''), 7.03 (d, *J* = 15.7 Hz, 1H, H2), 2.88 (s, 4H, H2') **(Supplementary Figure S7)**. ^{1}H NMR data are in accordance with the previous reports[48].

Homogenisation of post-mortem brain tissue. The post-mortem brain tissue samples were obtained from the Edinburgh Brain Bank, where they were flash-frozen and stored at −80 °C. Briefly, the tissue was homogenised using a VelociRuptor V2 Microtube Homogeniser (Scientific Laboratory Supplies, Cat. No. SLS1401, 5 meters/second, for two 20s cycles, 10s between cycles at 4 °C, in 10 volumes of ice-cold homogenisation buffer (10 mM Tris-HCl, 0.8 M NaCl, 1 mM EGTA, 0.1% Sarkosyl, 10% sucrose; pH 7.32) containing cOmplete™ Ultra Protease Inhibitor and PhosStop™ Phosphatase Inhibitor. The homogenate was centrifuged at 21,000 × g for 20 minutes at 4 °C, and the upper 90% of the supernatant was retained. The pellet was re-homogenised in 5 volumes of homogenisation buffer, then centrifuged at 21,000 × g for 20 minutes at 4 °C. The upper 90% of this supernatant was then removed and combined with the first supernatant, and aliquoted and frozen at -80 °C until used for further experiments. Total protein concentration was determined using a BCA assay (Thermo Fisher, Cat. No. 23227) as per the manufacturer's instructions.

Detailed experimental protocol for Simoa reagent preparation and plate preparation

Simoa magnetic bead coupling. Antibody-bead conjugation was performed as per manufacturer's instructions using Quanterix reagents. Briefly, paramagnetic carboxylated beads were vortexed for 30 sec and left on a rotator to mix gently for at least 10 min. The beads were then washed three times with the Bead Wash Buffer followed two times with Bead Conjugation Buffer. EDC (0.3 mg/mL) was used to activate the beads by placing the mixture in a rotator at 2-8°C for 30 min. The beads were once again washed with the Bead Conjugation Buffer. Buffer exchange of 100 µg of antibody was performed with a 50 kDa Amicon Ultra Spin Column device to change the storage buffer of the antibody to the Bead Conjugation Buffer. The concentrated antibody was recovered to ~ 100 µL volume and the antibody concentration was measured by nanodrop using A280. The antibody was diluted to 0.2 mg/mL in 300 µL of ice-cold bead conjugation buffer and used to resuspend the washed beads. The mixture was placed on a rotator at 2-8°C for 120 min. The antibody-coated beads were then washed twice with Bead Wash Buffer and antibody coating efficiency was determined by measuring the residual antibody concentration in the solution and supernatant of the first wash. Bead Blocking Buffer was used to block the beads with 45 min incubation on a rotator. Finally, the beads were washed once with Bead Wash Buffer followed by two washes with Bead Diluent Buffer and stored as a pellet at 4°C until use.

Simoa plate preparation. Simoa plates were prepared in a "3-step-assay" following the Quanterix protocol. A total volume of 100 µL of each sample, diluted with either Quanterix Homebrew Detector/Sample Diluent, diluent A, B, C, D, E from the sample diluent kit or Tau 2.0 sample diluent to the desired concentration, were added to a Simoa 96-Well Plate. Approximately 500k antibody-conjugated beads were added to each well and incubated on a shaker at 30°C and 800 rpm for 30 min (or 60 min for the 4B12 assay). All washing steps were performed using the automated 3-step-assay program on the Simoa Microplate Washer. The assay wells were washed three times with Simoa wash buffer A using Simoa Microplate Washer with magnet. To each well, 100 µL of biotinylated detector antibody (0.3/0.5/1 µg/mL, in Detector/Sample Diluent) was added and incubated on a shaker at 30°C and 800 rpm for 10 min (or 30 min) followed by three washes with Simoa wash buffer A using Simoa Microplate Washer with magnet. SBG (streptavidin β-galactosidase) was diluted to a concentration of 150 pM (or 50 pM, 300 pM) with SBG Dilution Buffer and 100 µL of the solution was added to the assay wells. The microplate was once again incubated on a shaker at 30°C and 800 rpm for 10 min followed by three washes with Simoa Wash buffer A using the Simoa Microplate Washer with magnet. Two final washes were performed with Simoa Wash Buffer B and 1 min incubation on a shaker. Finally, the buffer in the assay wells was completely removed and the beads were left to dry for 10 min and processed on the Quanterix SR-X™ Instrument. We avoid any sample or calibrant concentrations where $f_{ON} > 0.9$ in order to remain in a digital regime.

Simoa processing. The following steps were automated by using a Quanterix SR-X™ Instrument. The beads were resuspended using pre-warmed RGP (resorufin β-D-galactopyranoside) substrate. These beads suspensions were then transferred to the flow cells on the Simoa Disc. A low-power vacuum pulls the bead suspension into the disc channel, allowing the bead suspension to flow over an array of more than 200 thousand microwells, each large enough to hold a single bead. Oil was used to remove the excess beads on the surface of the array and seal the loaded wells. Then the wells were imaged, with those containing beads with at least one immunocomplex attached would giving fluorescence signals, whilst the plain beads did not. The fraction of beaded wells with enzyme activity (f_{ON}) was then reported by the machine.

Simoa data analysis. The readout of SIMOA assays is presented in terms of the fraction of beaded wells with enzyme activity (f_{ON}). This is subsequently used to calculate the average enzymes per bead (AEB). When $f_{ON} < 0.7$ (i.e. with less than 70% active beads), AEB is calculated in digital mode, assuming Poisson statistics for the number of captured molecules per "on" bead. In this case, AEB is determined by:

$$\mathrm{AEB}_{\mathrm{digital}} = -\ln\left[1 - f_{\mathrm{ON}}\right]$$

As for the wells with $f_{ON} > 0.7$, uncertainty rapidly grows to unacceptable levels for the Poisson distribution, the AEB is then switched to analog mode based on the intensity of a single enzyme via:

$$\mathrm{AEB}_{\mathrm{analog}} = \frac{f_{\mathrm{ON}} \times I_{\mathrm{bead}}}{I_{\mathrm{single}}}$$

where:

$$I_{\mathrm{single}} = \frac{f_{\mathrm{ON}} \times I_{\mathrm{bead}}}{-\ln\left[1 - f_{\mathrm{ON}}\right]} \quad \text{in arrays where } f_{\mathrm{ON}} < 0.1$$

We note however that for these assays, aggregates are being detected which may be bound by more than one enzyme. This means that the analog mode for calculating AEB cannot be used, and we revert to using digital mode. We avoid any sample or calibrant concentrations where $f_{ON} > 0.9$. Furthermore, while we use the term AEB (average enzyme per bead) for the purpose of consistency, in our case more than one enzyme can be bound per aggregate. After obtaining

the AEB values at each calibration level, a four-parameter logistic (4PL) curve was fitted (concentration of calibrators as x and AEB as y, and blanks were not included when establishing the calibration curve). The data points are weighted by 1 over during the fitting process.

$$y = D + \frac{A - D}{1 + (\frac{x}{C})^B}$$ (4PL equation)

Supplementary figures

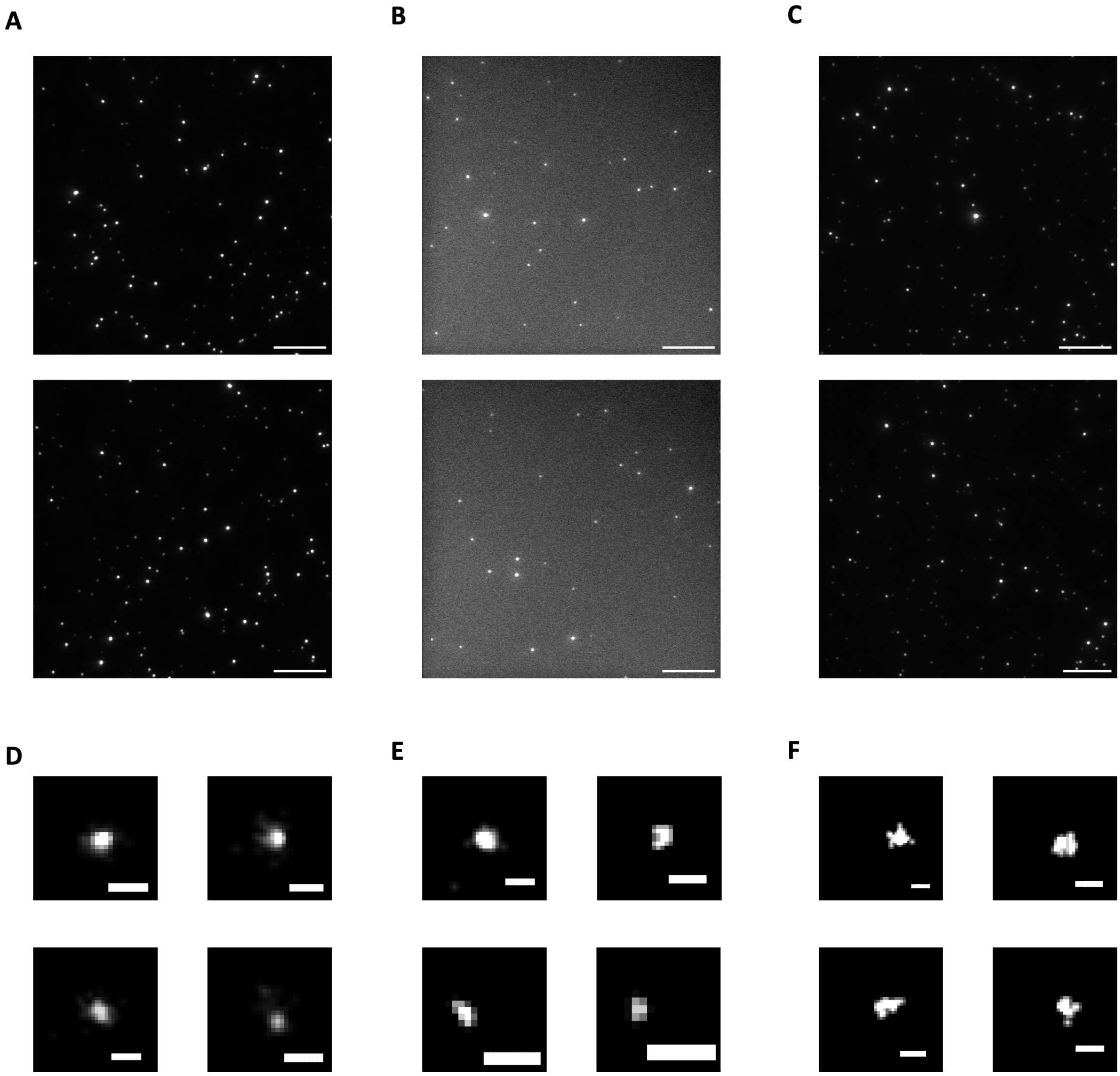

Supplementary Figure S1: Diffraction limited and super resolution images of coupled silica nanoparticles showing no significant aggregation. Diffraction limited images of (A) α-syn calibrators (15 nm silica nanoparticle) at 10 nM detected using SC211-SiMPull. **(B)** Aβ42 calibrators (15 nm silica nanoparticle) at 10 nM detected using 6E10-SiMPull. **(C)** tau calibrators (30 nm silica nanoparticle) at 1 nM detected using HT7-SiMPull. Representative images of super-resolved calibrators of **(D)** α-syn calibrators (15 nm silica nanoparticle), **(E)** Aβ42 calibrators (15 nm silica nanoparticle) and **(F)** tau calibrators (30 nm silica nanoparticle). Panel A-C: scale bar = 10 µm. Panel D-F: scale bar = 100 nm,

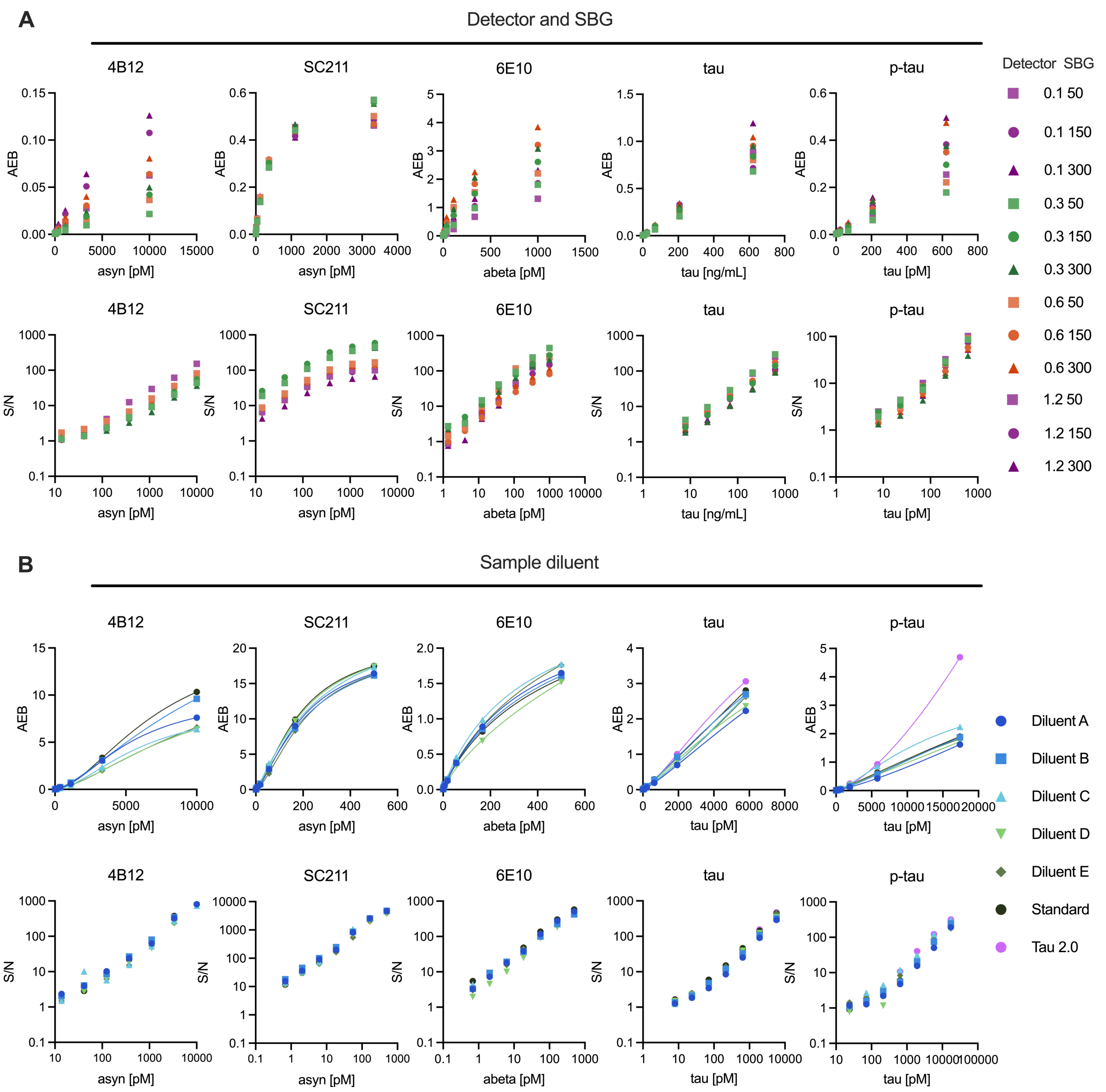

Supplementary Figure S2: Optimisation of the assay conditions (detector and SBG concentration) for enhanced performance. (A) The detector and SBG concentration were tested in a wide range for all assays across the concentration range for each assay to optimise the AEB and signal to noise ratio. The detector concentrations ranged from 0.1 to 1.2 ng/mL and the SBG concentration from 50 -150 pM. **(B)** Optimisation of sample diluent testing commercially available diluents.

S3

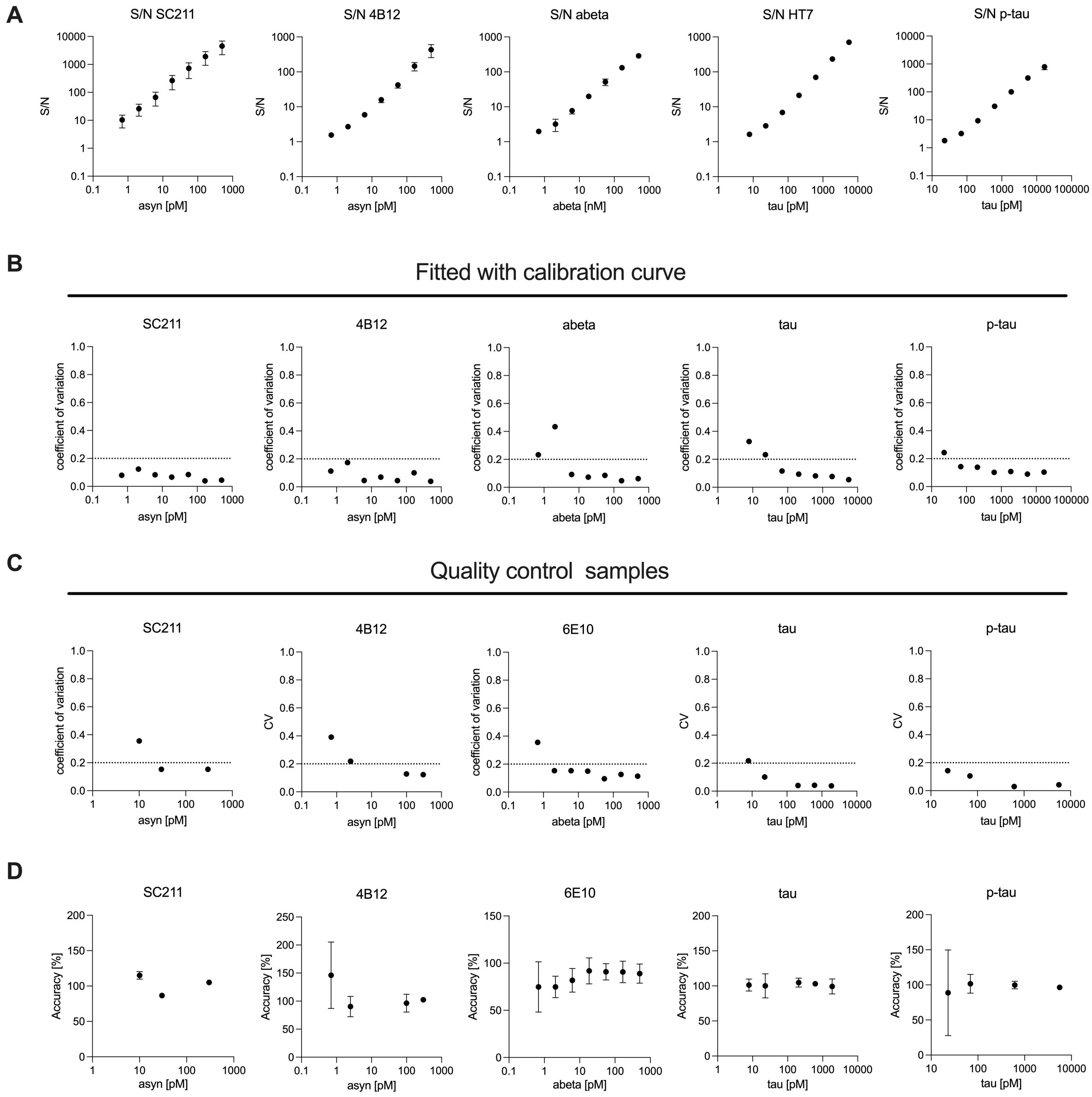

Supplementary Figure S3: Validation of signal to noise, coefficient of variation and accuracy of the Simoa aggregate assays. The assays were validated across four technical replicates across two days, containing the standard and five technical replicates of independently prepared samples to obtain the **(A)** Signal to noise across the calibration range of each assay for the optimised assay conditions, **(B)** the coefficient of variation after normalisation to the calibration curve, (C) the coefficient of variation from independently prepared samples and **(D)** their accuracy.

S4

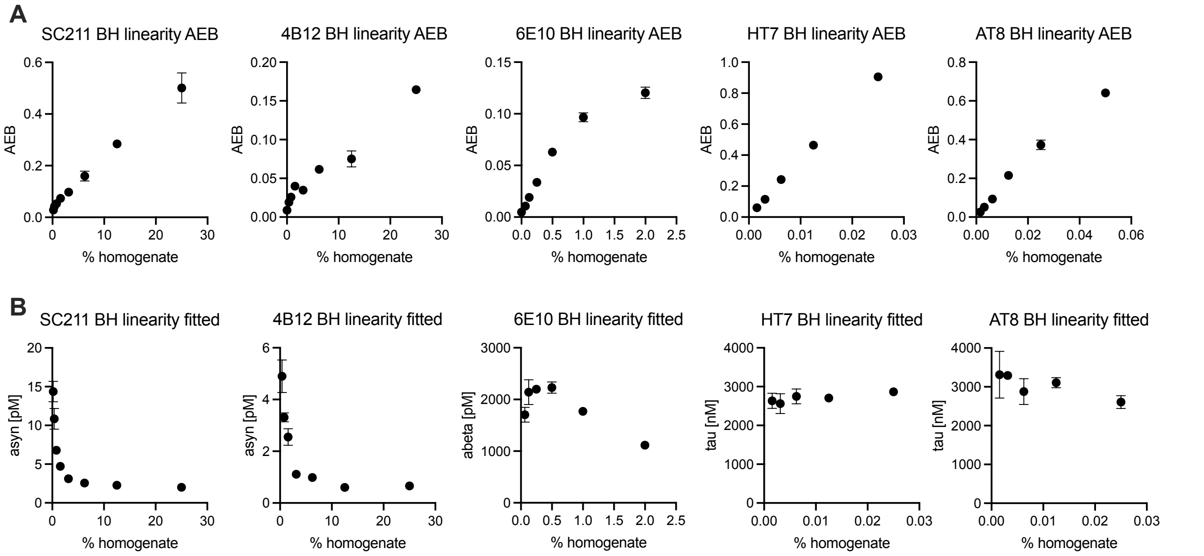

Supplementary Figure S4: Linearity test on brain homogenate sample. The linearity of the signal over a range of dilutions was ensured for all assays. (A) The AEB readout was obtained across a range of dilutions and fitted using a calibration curve to **(B)** back calculate the levels of the protein aggregates.

S5

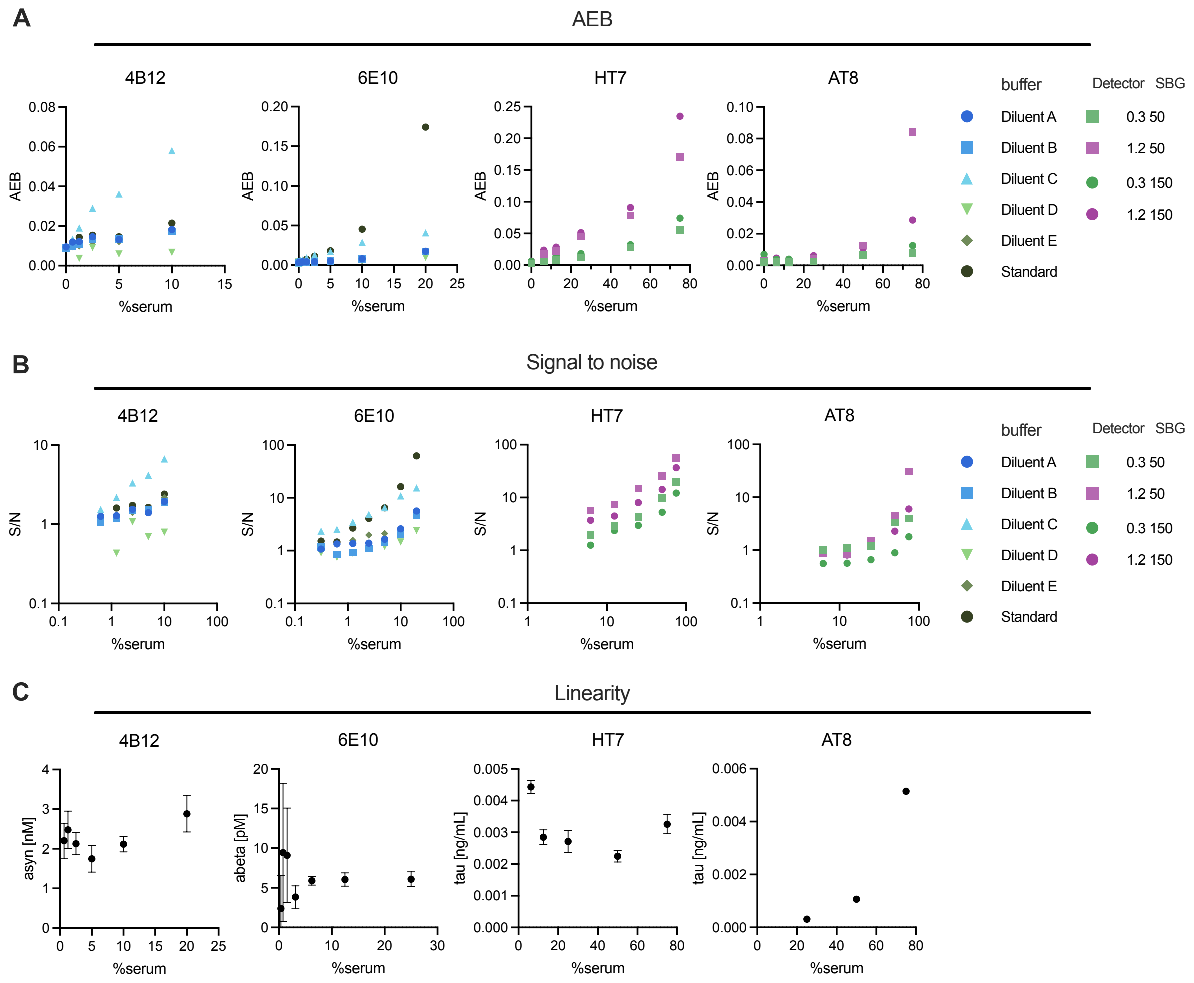

Supplementary Figure S5: Assay optimisation and linearity for human serum samples. The assay conditions (diluent, SBG and detector antibody concentration) were optimised specifically for serum samples and the linearity of the signal over a range of dilutions was ensured. **(A)** The assay conditions (detector: detector antibody concentration [µg/mL], SBG: SBG enzyme concentration [pM]) of the tau assays were optimised, as well as the sample diluent type for the 6E10 and 4B12 assays. **(B)** The conditions were selected to obtain the highest signal-to-noise ratio, specifically at low tau concentration. **(C)** The linear range of the serum samples across the concentration range was ensured by back calculating the fitted concentrations.

S6

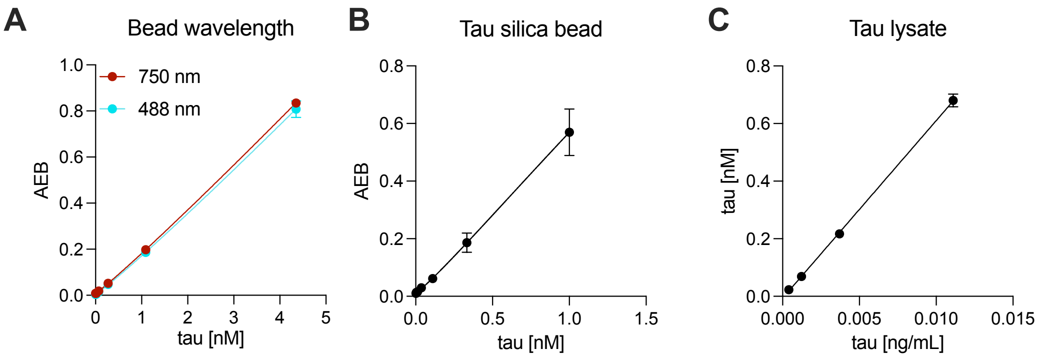

Supplementary Figure S6: Development of tau aggregate assay. (A) Comparison of tau-venus aggregates detected using the AT8 capture antibody coupled to 750-nm-fluorescent or 488-nm-fluorescent magnetic beads. **(B)** Curve of tau silica bead calibrant. (C) Determination of the concentration of tau aggregates in tau lysate standard.

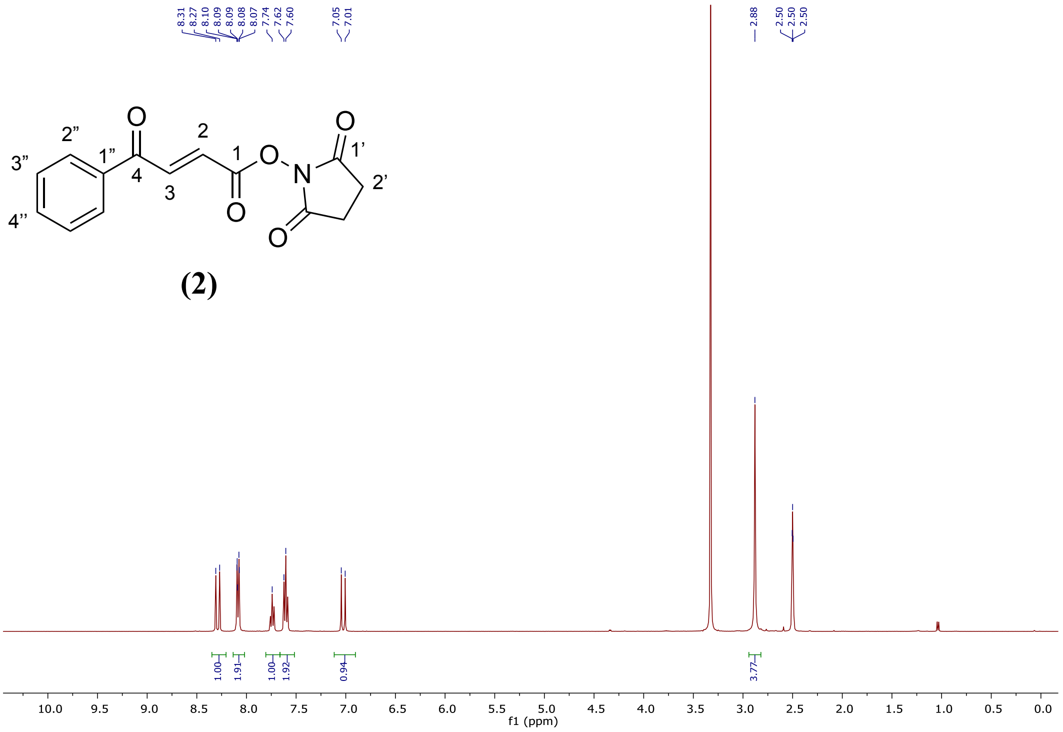

Supplementary Figure S7: ^{1}H spectrum of compound **2**.

Supplementary Table S1. Optimised conditions and limits of detection for aggregate assays. * indicates assay conditions for serum sample specific assays.

	4B12-4B12	SC211-SC211	6E10-6E10	HT7-HT7	AT8-AT8
Epitope	AA103-108	AA121-125	AA3-8	AA159-163	pS202 + pT205
Sample diluent	Diluent C	Diluent C	Homebrew	Tau 2.0	Tau 2.0
[Detector]	1 µg/mL	0.5 µg/mL	0.3 µg/mL	0.3 µg/mL (1.2 µg/mL)*	0.3 µg/mL (1.2 µg/mL)*
[SBG]	50 pM	50 pM	150 pM	150 pM (50 pM)*	150 pM (50 pM)*
Sample incubation	60 min, 30°C, 800 rpm	30 min, 30°C, 800 rpm	30 min, 30°C, 800 rpm	30 min, 30°C, 800 rpm	30 min, 30°C, 800 rpm
LoD	4.2 pM	0.625 pM	0.92 pM	17 pM	37 pM

Supplementary Table S2: Patient information brain homogenate.

**Patient**	**Sex**	**Age [years]**	**Braak Stage**	**Region**	**BBN**
**AD1**	Female	72	VI	BA6/8	BBN001.36924
**AD2**	Male	75	VI	BA6/8	BBN001.37400
**AD3**	Female	85	VI	BA6/8	BBN_25739
**AD4**	Male	75	VI	BA6/8	BBN001.36839
**AD5**	Male	75	VI	BA6/8	BBN001.36689
**HC1**	Female	71	0	BA6/8	BBN001.29882
**HC2**	Male	72	0	BA6/8	BBN001.30178
**HC3**	Female	73	0	BA6/8	BBN001.35138
**HC4**	Male	71	0	BA6/8	BBN001.30916
**HC5**	Male	82	0	BA6/8	BBN001.35549s

Supplementary Table S3. Results of aggregate measurements on AD and control serum. Values shown in unit of pM in format of mean (S.D.).

**Assay**	**All subjects**	**AD**	**Control**	**P value (t-test)**
SC211	24 (70)	4 (6)	44 (94)	0.12
4B12	230 (804)	387 (1112)	73 (199)	0.22
6E10	18 (58)	35 (79)	1 (3)	0.061
HT7	48 (46)	61 (56)	35 (29)	0.071
AT8	106 (203)	188 (263)	24 (29)	0.0081

Supplementary Table S4: Patient information of serum samples and aggregate measurements in [pM].

SUBJECT LABEL	AGE	GENDER	TYPE	4B12	SC211	6E10	HT7	AT8
**1**	66	f	AD	5.600	0.690	0.370	140.682	187.949
**2**	75	m	AD	2729.500	0.000	266.135	102.081	805.140
**3**	74	m	AD	13.590	5.590	0.590	61.035	23.442
**4**	67	f	AD	1.690	5.985	1.400	67.151	10.867
**5**	80	m	AD	0.030	0.000	0.285	84.395	187.949
**6**	64	f	AD	3.845	0.000	0.000	29.643	187.949
**7**	73	m	AD	425.530	0.000	89.295	61.035	19.978
**8**	84	m	AD	10.570	22.720	6.990	69.371	809.123
**9**	68	m	control	0.000	0.000	0.410	25.477	14.954
**10**	71	m	control	13.675	37.695	0.670	25.910	16.977
**11**	74	f	control	8.660	2.060	0.010	9.542	15.451
**12**	71	m	control	91.460	27.590	0.495	1.882	5.474
**13**	59	f	control	1.940	43.295	0.180	17.881	6.188
**14**	66	f	AD	2.075	0.000	0.050	44.357	6.336
**15**	74	m	control	0.820	0.000	0.805	27.652	6.596
**16**	77	m	control	25.935	41.955	1.600	48.714	55.292
**17**	49	f	control	15.365	1.545	0.195	36.703	1.195
**18**	57	f	control	3.130	0.445	0.000	36.394	73.642
**19**	75	m	control	0.000	0.000	0.000	111.330	15.027
**20**	79	f	control	1.860	0.000	0.000	23.688	0.000
**21**	71	m	AD	129.560	5.430	21.815	245.978	170.484
**22**	80	m	AD	0.000	0.000	0.000	13.930	20.853
**23**	70	m	control	0.000	1.085	0.000	89.080	12.255
**24**	75	m	control	16.560	17.150	0.020	19.922	40.050
**25**	72	f	AD	0.985	4.430	0.840	8.994	5.782
**26**	66	f	control	0.080	1.360	0.105	32.254	16.934
**27**	68	f	control	123.385	89.865	0.050	14.227	94.522
**28**	82	m	AD	9.700	0.000	38.705	66.046	0.000
**29**	63	f	control	216.420	244.190	0.355	45.948	76.709
**30**	62	f	control	886.530	0.380	0.000	84.384	8.037
**31**	80	m	AD	0.000	0.000	0.480	51.792	50.038
**32**	74	f	AD	24.575	6.615	0.265	13.199	12.953
**33**	76	m	control	0.000	0.000	0.005	11.328	0.000
**34**	53	m	control	43.465	366.845	13.960	5.959	6.310
**35**	86	f	control	0.040	9.305	0.000	32.151	4.886
**36**	75	m	AD	4.465	0.000	15.650	20.885	136.036
**37**	68	m	AD	4339.560	12.085	252.170	8.204	460.484
**38**	76	f	AD	0.000	0.000	0.000	13.149	13.324
**39**	59	m	AD	5.240	1.760	4.645	48.147	63.188
**40**	79	f	AD	37.020	10.965	6.245	70.625	587.099
